# Obesity Does Not Increase Perioperative Outcomes in Older Patients Undergoing Thoracoscopic Anatomic Lung Cancer Surgery

**DOI:** 10.3389/fonc.2022.881467

**Published:** 2022-05-06

**Authors:** Chaoyang Tong, Tingting Li, Yaofeng Shen, Hongwei Zhu, Jijian Zheng, Jingxiang Wu

**Affiliations:** ^1^Department of Anesthesiology, Shanghai Chest Hospital, Shanghai Jiao Tong University, Shanghai, China; ^2^Department of Anesthesiology, Shanghai Children’s Medical Center, School of Medicine, Shanghai Jiao Tong University, Shanghai, China

**Keywords:** elderly, obesity, thoracoscopic surgery, lung cancer, outcomes

## Abstract

**Objectives:**

To investigate the relationship between obesity status and perioperative outcomes in elderly patients undergoing thoracoscopic anatomic lung cancer surgery.

**Methods:**

From January 2016 to December 2018, we performed a monocentric retrospective cohort study among 4164 consecutive patients aged 65 years or older who underwent thoracoscopic anatomic lung cancer surgery at Shanghai Chest Hospital. Two groups were stratified by body mass index (BMI): nonobese (BMI<28kg/m^2^) and obese status (BMI≥28kg/m^2^). Using a 1:1 propensity score matching (PSM) analysis to compare perioperative outcomes between two groups.

**Results:**

4035 older patients were eventually enrolled, with a mean age of 69.8 years (range: 65-87), and 305 patients were eligible for obese status, with a mean BMI of 29.8 ± 1.7kg/m^2^. Compared with nonobese patients, obese patients were more likely to have higher rates of intraoperative hypoxemia (1.2% vs 3.9%, P=0.001) and new-onset arrhythmia (2.3% vs 4.3%, P=0.034). The difference in intraoperative transfusion and conversion rates and postoperative outcomes regarding pulmonary complications, new-onset arrhythmia, transfusion, length of hospital stay, 30-day readmission and hospitalization costs between two groups were not significant (P>0.05). After a 1:1 PSM analysis, the difference in both intraoperative and postoperative complications among two groups were not significant (P>0.05). In subgroup analysis, patients with BMI≥30kg/m^2^ had a similar incidence of perioperative complications compared to patients with BMI between 28 and 30 kg/m^2^ (P>0.05).

**Conclusions:**

Our research data support evidence for “obesity paradox” and also contribute the growing body of evidence that obesity in older patients should not exclude candidates for thoracoscopic anatomic lung cancer surgery.

## Introduction

Over the last few decades, the prevalence of obesity has shifted dramatically (1, 2), and obesity-related metabolic diseases such as hypertension, diabetes and hyperlipidemia have also gradually increased (3–5), becoming a worldwide health problem. Currently, obesity-related cancer rates have become a focus of concern, although the effect varies widely depending on the types of cancer (4, 6–9). Among these types, an inverse relationship between obesity and lung cancer risk has been reported in previous studies (6–9). And the biological mechanisms underlying the major role of obesity in chronic inflammation and carcinogenesis have also been extensively evaluated (4, 10–12).

Indeed, the proportion of overweight and obese lung cancer patients undergoing lung surgery has increased during the last decades (13, 14). Besides, obesity-related preoperative comorbidities increase the risk of perioperative surgical complications, mutually reinforcing (15–17). Additionally, obesity characterized by substantial girth and excess mediastinal fat has a profound impact on the technical issues during the procedure, especially in minimally invasive surgery (MIS) (18, 19). Moreover, with the aging of society, the influence of obesity on perioperative outcomes is further complicated by the specific attributes of elderly surgical patients, such as frailty, cognitive decline, impaired preoperative pulmonary function and tissue fragility (20, 21).

Recently, a retrospective study conducted by Tabatabai and colleagues showed that obesity class II-III had prognostic value in predicting increased rates of postoperative complications, prolonged length of hospital stay and no-home discharge locations in elderly patients undergoing spine, hip, and knee procedures (22). Up to now, there was no literature about the influence of obesity on perioperative outcomes in elderly patients during thoracoscopic anatomic lung cancer surgery. In the present study, by reviewing a large sample of clinical data, we aimed to evaluate the relationship of obesity to perioperative outcomes in thoracoscopic anatomic lung cancer surgery.

## Materials and Methods

### Study Design and Patients

**Figure 1:**
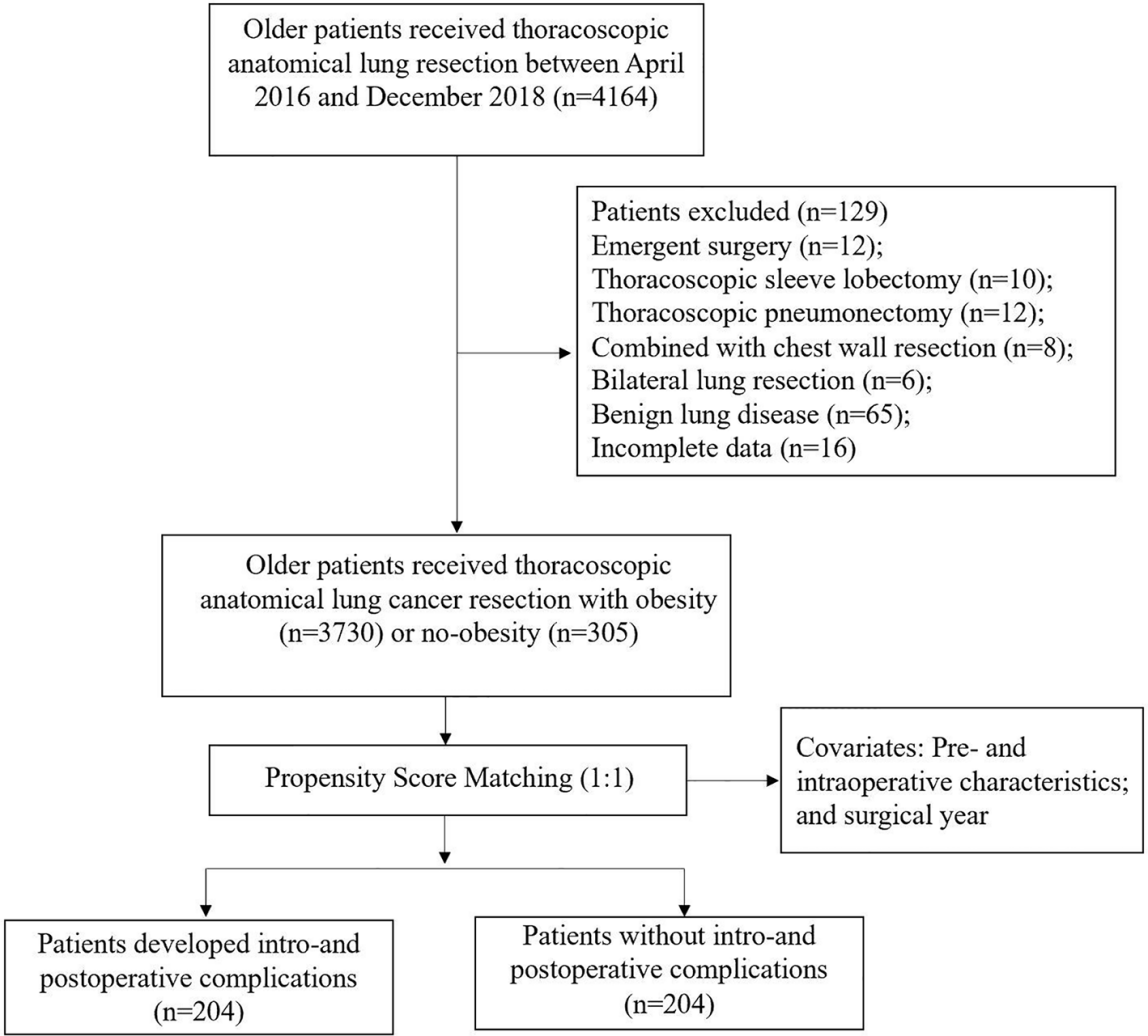
Patient flowchart.

From January 2016 to December 2018, we performed a monocentric retrospective cohort study based on a prospectively collected database, including 4164 consecutive patients aged 65 years or older who underwent thoracoscopic anatomic lung cancer surgery. Excluded patients were described in the flow diagram (**Figure 1**). 4035 elderly patients were enrolled in the final analysis. The Institutional Review Board (IRB) of Shanghai Jiao tong University, Shanghai Chest Hospital (IS21119) approved this study and waived the need for informed consent.

### Anesthesia Protocol

All patients were routinely monitored by electrocardiogram, pulse oximetry, non-invasive blood pressure (NIBP) and capnography. Radial artery intubation and right internal jugular central venous catheterization (CVP) were used to monitor invasive blood pressure (IBP). For thoracic paravertebral blockade (TPVB), 20 mL 0.5% ropivacaine was injected to the T4-T5 by the experienced anesthesiologist under the guidance of ultrasound before surgery. Intraoperative lung protective ventilation (LPV) strategies consisted of low-tide ventilation based on ideal body weight (≤8mL/kg), PEEP=5cmH2O, lung recruitment and maintenance of airway pressure <30cmH2O. Extubation was performed in the operating room or post anesthesia care unit (PACU) for all suitable patients. All patients treated with patient-controlled analgesia (PCA) pump, including sufentanil1.0 μg/kg + desoxocin 0.4mg/kg.

### Technique of Operation

Since 2016, the high-volume center of Shanghai Chest Hospital has performed nearly 10,000 lung operations annually, of which thoracoscopic surgery accounts for more than 80 percent. All possible thoracoscopic surgery was determined by the participating surgeons based on the individual patient’s preoperative evaluation. For all patients, thoracoscopic anatomic lung resection plus systematic lymph node dissection were considered as the best treatment for primary lung cancer.

### Data Collection and Definition

Perioperative clinical data were prospectively pooled from our institution’s electronic medical system, enrolling patient’s baseline and intraoperative characteristics, and perioperative outcomes including hypoxemia, transfusion, new-onset arrhythmia, conversion to thoracotomy, pulmonary complications, length of hospital stay (LOS), 30-day readmission and hospitalization costs. Hypoxemia was defined as SpO_2_<90% for more than 10 consecutive minutes. Postoperative pulmonary complications (PPCs) refer to the European Perioperative Clinical Outcome (EPCO) (23). According to the 2014 Guidelines of the American Association of Thoracic Surgeons (AATS) (24), perioperative new-onset arrhythmia included incidents of atrial fibrillation (AF) and atrial flutter. The 30-day readmission included in the analysis was an unplanned return to the hospital due to various postoperative complications.

We classified the obesity status according to the body mass index (BMI, weight in kilograms divided by height in meters squared) of the Guidelines for Prevention and Control of Overweight and Obesity in Chinese Adults (25). Two groups were stratified by BMI: nonobese (BMI<28kg/m^2^) and obese status (BMI≥28kg/m^2^). In subgroup analysis, the perioperative outcomes between patients with obesity class I (28kg/m^2^ ≤BMI<30kg/m^2^) and obesity class II (BMI≥30kg/m^2^) were investigated.

### Statistical Analysis

Data was tested for normal distribution with Q-Q plot. Continuous variables were compared between nonobese and obese patients using Two independent sample t-test or Mann-Whitney U test. Categorical variables were compared with Chi-square test or Fisher exact test, depending on the sample size. To reduce the selection bias and other potential confounding effects, we performed a 1:1 propensity score matching (PSM) analysis using a caliper size of 0.01 to compare perioperative outcomes between two cohorts. All pre-, intraoperative variables and surgical year were included in the PSM analysis. Standardized mean difference (SMD) between two cohorts on all covariables after matching was calculated, with differences of <10% indicating adequate balance in the matched cohort. Statistical analysis was performed using the SPSS 26.0 software (IBM Corp., Armonk, NY, USA). R version 4.1.2 was used with the tableone, ggplot2, reshape2, survey and Matching packages*. P*-value<0.05 was considered statistically significant.

## Results

### Study Cohort

**Figure 2:**
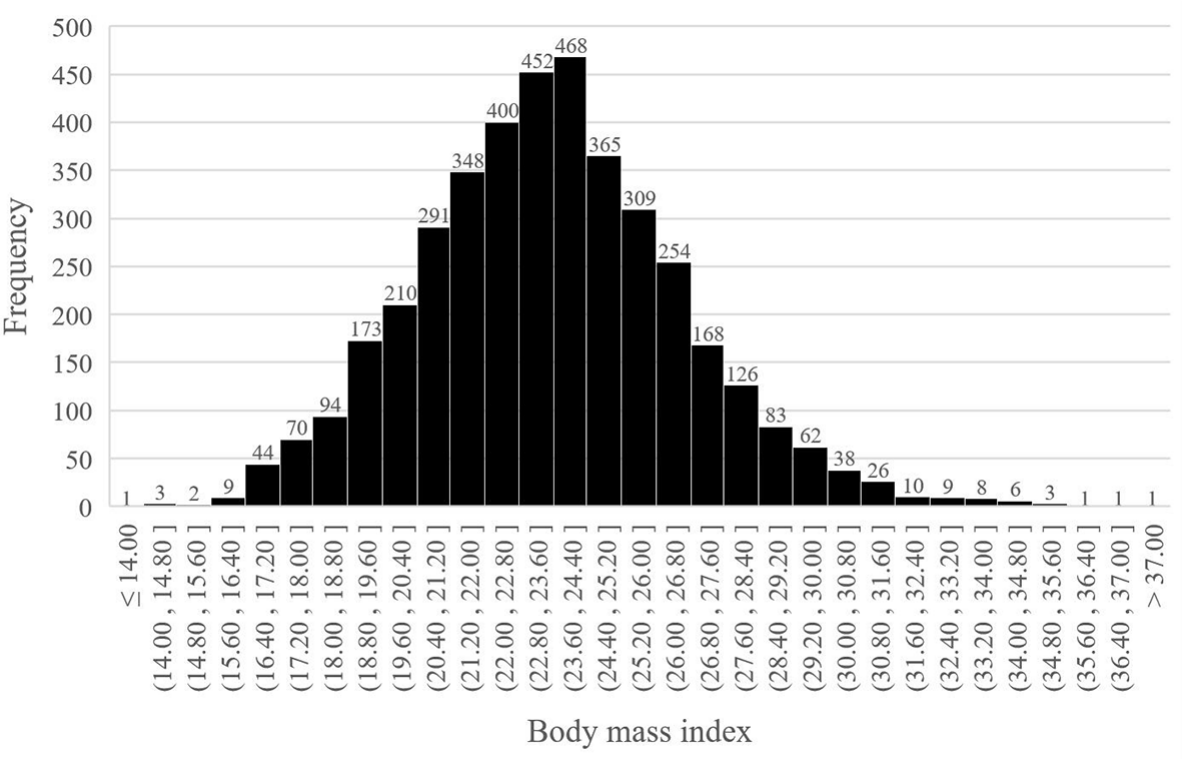
Body mass index distribution.

From January 2016 to December 2018, 4035 older patients with a mean age of 69.8 years (range: 65-87) underwent thoracoscopic anatomical lung cancer surgery, of which 17.6% (712 out of 4035) underwent segmentectomy resection and 82.4% (3323 out of 4035) underwent lobectomy resection, and 17.6% (305 out of 4035) were eligible for obese status, with a mean BMI of 29.8 ± 1.7kg/m^2^ (**Figure 1**). The BMI distribution of all enrolled patients were depicted in **Figure 2**.

Patients with obese status had a higher American Society of Anesthesiologists (ASA) classification (III/IV, 23.3% vs 16.3%, P=0.007), and increased rates of hypertension (16.1% vs 11.8%, P=0.03) and diabetes mellitus (10.2% vs 6.7%, P=0.022), and better preoperative lung function (FEV_1_/FVC, 105.2 ± 8.3 vs 100.9 ± 9.9, P<0.001; DLCO%, 96.7 ± 17.9 vs 90.5 ± 17.8, P<0.001) when compared with their counterparts. Additionally, patients developed with obese status required longer operative time (108.5 ± 40.1 vs 102.4 ± 39.5mins, P=0.009) compared with nonobese patients (**Tables 1**, **2**).

**Table 1:** Baseline characteristics stratified by BMI.

Variables^a^	BMI<28 (n = 3730)	BMI≥28 (n = 305)	P Value
Age, years	69.8 ± 4.1	69.5 ± 3.9	0.309
Sex			0.513
Male sex	1748 (46.9)	137 (44.9)	
Female sex	1982 (53.1)	168 (55.1)	
ASA classification			0.007^b^
I	108 (2.9)	8 (2.6)	
II	3014 (80.8)	226 (74.1)	
III/IV	608 (16.3)	71 (23.3)	
Comorbidity			
Hypertension	442 (11.8)	49 (16.1)	0.030^b^
Diabetes mellitus	250 (6.7)	31 (10.2)	0.022^b^
Coronary artery disease	45 (1.2)	5 (1.6)	0.585
Stroke/TIA	22 (0.6)	2 (0.7)	1.000
FEV_1_/FVC, %	100.9 ± 9.9	105.2 ± 8.3	<0.001^b^
DLCO%	90.5 ± 17.8	96.7 ± 17.9	<0.001^b^
Chemoradiotherapy	4 (0.1)	1 (0.3)	0.325
Tumor size, cm	2.1 ± 1.1	2.1 ± 1.1	0.791
Clinical tumor stage			0.349
T1a	522 (14.0)	33 (10.8)	
T1b	1577 (42.3)	146 (47.9)	
T1c	1013 (27.2)	74 (24.3)	
T2a	401 (10.8)	37 (12.1)	
T2b	135 (3.6)	11 (3.6)	
T3	67 (1.8)	3 (1.0)	
T4	15 (0.4)	1 (0.3)	
Advanced clinical stage (T≥2)	618 (16.6)	52 (17.0)	0.828

a Continuous data are shown as mean ± standard deviation and categoric data as number (%).

b Statistically significant (P < 0.05). BMI, Body mass index (kg/m^2^); ASA, American Society of Anesthesiology; TIA, Transient cerebral ischemic attack; FEV_1_, Forced expiratory volume in 1 second; FVC, Forced vital capacity; DLCO, Diffusion capacity for carbon monoxide.

**Table 2:** Intraoperative characteristics stratified by BMI.

Variables^a^	BMI<28 (n = 3730)	BMI≥28 (n = 305)	P Value
Lymph nodes calcification	288 (7.7)	27 (8.9)	0.479
Clinical nodal involvement	255 (6.8)	24 (7.9)	0.494
Pleural adhesions	130 (3.5)	15 (4.9)	0.196
Type of resection			0.168
Segmentectomy resection	667 (17.9)	45 (14.8)	
Lobectomy resection	3063 (82.1)	260 (85.2)	
Thoracoscopic resection			0.873
Uni-portal	328 (8.8)	26 (8.5)	
Multi-portal	3402 (91.2)	279 (91.5)	
Approach			0.743
VATS	3607 (96.7)	296 (97.0)	
RATS	123 (3.3)	9 (3.0)	
Anesthesia type			0.847
GA alone	3110 (83.4)	253 (83.0)	
GA plus TPVB	620 (16.6)	52 (17.0)	
Location of resection			0.459
Left	1452 (38.9)	106 (34.8)	
Left upper	929 (24.9)	74 (24.3)	
Left lower	523 (14.0)	32 (10.5)	
Right	2278 (61.1)	199 (65.2)	
Right upper	1218 (32.7)	109 (35.7)	
Right middle	334 (9.0)	27 (8.9)	
Right lower	726 (19.5)	63 (20.7)	
Ipsilateral reoperation	3 (0.1)	1 (0.3)	0.270
Operative time, mins	102.4 ± 39.5	108.5 ± 40.1	0.009^b^

a Continuous data are shown as mean ± standard deviation and categoric data as number (%).

b Statistically significant (P < 0.05). BMI, Body mass index (kg/m^2^); VATS, Video-assisted thoracoscopic surgery; RATS, Robotic-assisted thoracoscopic surgery; GA, General anesthesia; TPVB, Thoracic paravertebral blockade.

### Perioperative Outcomes Between Two Cohorts Before and After a 1:1 PSM

Compared with nonobese patients, the rates of intraoperative hypoxemia (1.2% vs 3.9%, P=0.001) and new-onset arrhythmia (2.3% vs 4.3%, P=0.034) were higher in obese patients (**Table 3**). The differences among intraoperative transfusion, conversion rates and postoperative outcomes including pulmonary complications, new-onset arrhythmia, transfusion, LOS, 30-day readmission and hospitalization costs were not significant (**Table 3**). After a 1:1 PSM analysis, all patient characteristics were comparable in two cohorts (**Tables 4**, **5**). We investigated perioperative outcomes in 408 patients (204 pairs), the differences in both intra- and postoperative complications were not significant (**Table 6**). In subgroup analysis, the perioperative outcomes between patients with obesity class I and obesity class II were explored. When all perioperative data were comparable (**Supplementary Tables S1, S2**), we found that higher severity of obesity did not increase the incidence of perioperative complications (**Supplementary Table S3**).

**Table 3:** Intra- and postoperative complications stratified by BMI.

Variables^a^	BMI<28 (n = 3730)	BMI≥28 (n = 305)	P Value
Intraoperative complications			
Hypoxemia	46 (1.2)	12 (3.9)	0.001^b^
Transfusion	18 (0.5)	1 (0.3)	1.000
New-onset arrhythmia	86 (2.3)	13 (4.3)	0.034^b^
Conversion to thoracotomy	91 (2.4)	6 (2.0)	0.605
Postoperative complications			
PPCs	1309 (35.1)	118 (38.7)	0.207
Atelectasis	29 (0.8)	4 (1.3)	0.310
Pulmonary infection	1293 (34.7)	117 (38.4)	0.193
Respiratory failure	14 (0.4)	2 (0.7)	0.627
New-onset arrhythmia	174 (4.7)	17 (5.6)	0.472
Transfusion	51 (1.4)	2 (0.7)	0.325
Length of hospital stay, day	5[4-6]	5[4-6]	0.669
30-day readmission	19 (0.5)	2 (0.7)	1.000
Hospitalization costs, USD	10057 ± 2655	10193 ± 2250	0.434

a Continuous data are shown as mean ± standard deviation and categoric data as number (%); Length of hospital stay, values as median [interquartile range].

b Statistically significant (P < 0.05). BMI, Body mass index (kg/m^2^); PPCs, Postoperative pulmonary complications; USD, United States dollar.

**Table 4:** Baseline characteristics stratified by BMI after a 1:1 PSM.

Variables^a^	BMI<28 (n = 204)	BMI≥28 (n = 204)	SDM	P Value
Age, years	69.0 ± 3.4	69.4 ± 3.8	0.100	0.282
Sex			0.069	0.484
Male sex	84 (41.2)	91 (44.6)		
Female sex	120 (58.8)	113 (55.4)		
ASA classification			0.022	0.857
I	4 (2.0)	5 (2.5)		
II	160 (78.4)	156 (76.5)		
III/IV	40 (19.6)	43 (21.1)		
Comorbidity				
Hypertension	17 (8.3)	28 (13.7)	0.072	0.082
Diabetes mellitus	19 (9.3)	18 (8.8)	0.017	0.863
Coronary artery disease	3 (1.5)	3 (1.5)	<0.001	1.000
Stroke/TIA	0 (0)	2 (1.0)	0.014	0.499
FEV_1_/FVC, %	104.8 ± 8.7	104.5 ± 7.9	0.042	0.672
DLCO%	94.2 ± 16.9	95.7 ± 17.5	0.087	0.381
Chemoradiotherapy	0 (0)	1 (0.5)	0.099	1.000
Tumor size, cm	2.0 ± 1.1	2.1 ± 1.1	0.079	0.425
Clinical tumor stage			0.082	0.449
T1a	36 (17.6)	26 (12.7)		
T1b	95 (46.6)	95 (46.6)		
T1c	45 (22.1)	49 (24.0)		
T2a	15 (7.4)	24 (11.8)		
T2b	9 (4.4)	7 (3.4)		
T3	4 (2.0)	2 (1.0)		
T4	0 (0)	1 (0.5)		
Advanced clinical stage (T≥2)	28 (13.7)	34 (16.7)	0.082	0.408

a Continuous data are shown as mean ± standard deviation and categoric data as number (%). BMI, Body mass index (kg/m^2^); PSM, Propensity score matching; SMD, Standardized mean difference; ASA, American Society of Anesthesiology; TIA, Transient cerebral ischemic attack; FEV_1_, Forced expiratory volume in 1 second; FVC, Forced vital capacity; DLCO, Diffusion capacity for carbon monoxide.

**Table 5:** Intraoperative characteristics stratified by BMI after a 1:1 PSM.

Variables^a^	BMI<28 (n = 204)	BMI≥28 (n = 204)	SMD	P Value
Lymph nodes calcification	17 (8.3)	17 (8.3)	<0.001	1.000
Clinical nodal involvement	16 (7.8)	16 (7.8)	<0.001	1.000
Pleural adhesions	14 (6.9)	11 (5.4)	0.061	0.536
Type of resection			0.014	0.885
Segmentectomy resection	28 (13.7)	27 (13.2)		
Lobectomy resection	176 (86.3)	177 (86.8)		
Thoracoscopic resection			0.010	0.307
Uni-portal	16 (7.8)	22 (10.8)		
Multi-portal	188 (92.2)	182 (89.2)		
Approach			0.058	0.241
VATS	196 (96.1)	200 (98.0)		
RATS	8 (3.9)	4 (2.0)		
Anesthesia type			0.026	0.199
GA alone	180 (88.2)	171 (83.8)		
GA plus TPVB	24 (11.8)	33 (16.2)		
Location of resection			0.044	0.912
Left	73 (35.8)	74 (36.3)		
Left upper	49 (24.0)	54 (26.5)		
Left lower	24 (11.8)	20 (9.8)		
Right	131 (64.2)	130 (63.7)		
Right upper	64 (31.3)	68 (33.3)		
Right middle	23 (11.3)	20 (9.8)		
Right lower	44 (21.6)	42 (20.6)		
Ipsilateral reoperation	1 (0.5)	0 (0)	0.099	1.000
Operative time, mins	108.4 ± 37.8	108.1 ± 40.0	0.009	0.927

a Continuous data are shown as mean ± standard deviation and categoric data as number (%). BMI, Body mass index (kg/m^2^); PSM, Propensity score matching; SMD, Standardized mean difference; VATS, Video-assisted thoracoscopic surgery; RATS, Robotic-assisted thoracoscopic surgery; GA, General anesthesia; TPVB, Thoracic paravertebral blockade.

**Table 6:** Intra- and postoperative complications stratified by BMI after a 1:1 PSM.

Variables^a^	BMI<28 (n = 204)	BMI≥28 (n = 204)	P Value
Intraoperative complications			
Hypoxemia	4 (2.0)	10 (4.9)	0.103
Transfusion	0 (0)	1 (0.5)	1.000
New-onset arrhythmia	4 (2.0)	9 (4.4)	0.159
Conversion to thoracotomy	5 (2.5)	3 (1.5)	0.724
Postoperative complications			
PPCs	74 (36.3)	79 (38.7)	0.609
Atelectasis	2 (1.0)	3 (1.5)	1.000
Pulmonary infection	73 (35.8)	78 (38.2)	0.608
Respiratory failure	0 (0)	2 (1.0)	0.499
New-onset arrhythmia	9 (4.4)	11 (5.4)	0.647
Transfusion	1 (0.5)	0 (0)	0.100
Length of hospital stay, day	5[4-7]	5[4-6]	0.104
30-day readmission	2 (1.0)	1 (0.5)	1.000
Hospitalization costs, USD	10030 ± 2516	9966 ± 2069	0.787

a Continuous data are shown as mean ± standard deviation and categoric data as number (%); Length of hospital stay, values as median [interquartile range]. BMI, Body mass index (kg/m^2^); PSM, Propensity score matching; PPCs, Postoperative pulmonary complications; USD, United States dollar.

## Discussion

305 older patients who underwent thoracoscopic anatomical lung cancer surgery were eligible for obese status. Our study found that elderly obese patients had similar rates of perioperative complications compared to nonobese patients, and that the severity of obesity was unlikely to increase the incidence of adverse outcomes. Therefore, obesity status among elderly patients should not be a hindrance to preoperative evaluation and surgical planning during thoracoscopic anatomic lung cancer surgery.

The World Health Organization (WHO) (26) has recommended BMI cutoff points for underweight(<18.5kg/m^2^), normal weight (18.5 to 24.9kg/m^2^), overweight (25 to 29.9 kg/m^2^) and obesity (>30kg/m^2^) to predict health risks, including risk of all cancer types and non-cancer diseases. However, whether the criteria applied to Asian populations remains controversial (25, 27, 28). The BMI classification criteria for obesity in this investigation by referring to the Guidelines for Prevention and Control of Overweight and Obesity in Chinese Adults, which may be more suitable for clinical studies in Chinese population.

MIS has been established to improve perioperative adverse outcomes, especially in patients at high risk such as elderly and obese patients, with regard to the standard open approaches (13, 29). Presumably, the proportion of elderly patients with elevated BMI undergoing thoracic surgery will constantly increase in the future (13, 14, 19). Intuitively, it seems that longer operative time, reduced mobility, impaired diaphragm movement, and obesity-related comorbidities should be associated with an increased risk of lung resection. Therefore, it is mandatory to fully understand the influence of obesity on perioperative outcomes of elderly patients.

In terms of intraoperative complications, although before a 1:1 PSM, obese patients developed higher rates of hypoxemia and new-onset arrhythmia, but had comparable transfusion, conversion rates and operative time compared with nonobese patients, and none of these differences were significant after PSM. Our previous published literature echoed these results and did not find that elevated BMI was associated with high rates of intraoperative conversion and new-onset arrhythmia (30, 31). Similarly, Guerrera has evaluated the impact of morbidly obesity on perioperative clinical outcomes after thoracoscopic lobectomy and found that obese patients did not increase conversion rates, blood loss and surgical time (32). Conversely, a few studies have shown that obesity was associated with increased operative time (33).

Our research results may also provide the evidence for the “obesity paradox” in elderly patients undergoing thoracoscopic anatomic lung cancer surgery, which has been widely reported in published documents (14–17). The postoperative complications regarding pulmonary complications, new-onset arrhythmia, transfusion, LOS, 30-day readmission and hospitalization costs were not significant between obese and nonobese patients. Ferguson et al. showed that being overweight or obese did not increase the risk of postoperative complications in any category after major lung resection (34). Also, Thomas et al. conducted a retrospective cohort study of 19,635 patients undergoing lobectomy for primary lung cancer and concluded that obesity was not associated with increased incidence of postoperative complications, except for cardiovascular complications and had a statistical protective effect regarding surgical complications (35). Moreover, a systematic review with meta-analysis demonstrated that overall morbidity and in-hospital mortality were significantly decreased in obese patients (36).

Our investigation also assessed the effect of obesity severity on perioperative outcomes in older patients and found that the difference was not significant between the obesity class I and obesity class II. And the results were consistent with other published researches (14, 32). A retrospective study conducted by Williams using Society of Thoracic Surgeons General Thoracic Surgery Database indicated that overweight and obese class I to II patients had a lower risk of pulmonary complications and any postoperative events (14). In contrast to the results of our present study, Zogg (37) and De Oliveira’s (38) research showed that obese class II and III patients experienced marginally increased odds of morbidity and an increased risk of pulmonary complications, respectively. The variability of results may be related to the fact that high BMI does not distinguish between body composition phenotypes that have important effects on surgical risk and outcomes (39). Relying on BMI augmentation categories alone to assess the surgical risk of major lung resection is fraught with challenges.

Potential shortcomings of this study include as follows. First, as a retrospective study based on a prospectively collected database, it has the inherent design biases. Besides, single-institute study has specific generalization limitations. Second, due to the limited granularity of postoperative care data, some poor outcomes, such as pain control (40), other surgical complications and mortality, could not be pooled in this study. Third, the relationship between obesity and long-term outcomes needs further investigation (41, 42).

## Conclusions

By conducting a monocentric retrospective cohort study of 4035 elderly patients receiving thoracoscopic anatomic lung cancer surgery, our study found that obesity status and obesity severity dose not increase perioperative adverse outcomes. Even among older patients, these data support evidence for “obesity paradox”. These data also contribute the growing body of evidence that obesity in older patients should not exclude candidates for thoracoscopic anatomic lung cancer surgery.

## Data Availability Statement

The original contributions presented in the study are included in the article/**Supplementary Material**. Further inquiries can be directed to the corresponding authors.

## Ethics Statement

This study was approved by the Institutional Review Board at Shanghai Chest Hospital (IS21119), and the informed consent was waived because of the retrospective nature of the study.

## Author Contributions

CT, TL and YS: study conception, design, statistic analysis and drafting of the manuscript. CT, YS and HZ: acquisition of data. CT, JZ and JW: analysis and interpretation of data. JWand JZ: critical revision. All authors contributed to the article and approved the submitted version.

## Funding

This work was supported by National Natural Science Foundation of China (82071233) and Shanghai Shen Kang Hospital Development Center Project (SHDC2020CR4063).

## Conflict of Interest

The authors declare that the research was conducted in the absence of any commercial or financial relationships that could be construed as a potential conflict of interest.

## Publisher’s Note

All claims expressed in this article are solely those of the authors and do not necessarily represent those of their affiliated organizations, or those of the publisher, the editors and the reviewers. Any product that may be evaluated in this article, or claim that may be made by its manufacturer, is not guaranteed or endorsed by the publisher.
