# Supplementary material for: Obesity Does Not Increase Perioperative Outcomes in Older Patients Undergoing Thoracoscopic Anatomic Lung Cancer Surgery
Source: Front Oncol. 2022 May 6;12:881467. doi: 10.3389/fonc.2022.881467 (PMC9121795; doi:10.3389/fonc.2022.881467)
Supplement: Supplementary file 1 [file Table_1.docx]

Supplementary table S1 Baseline characteristics stratified by BMI

| Variables ^a^ | 28≤BMI<30 kg/m^2^  (n=202) | | BMI≥30 kg/m^2^  (n=103) | | *P* Value | |
| --- | --- | --- | --- | --- | --- | --- |
| Age, years | 69.4±4.0 | | 69.9±3.8 | | 0.276 | |
| Sex |  | |  | | 0.200 | |
| Male sex | 96(47.5) | | 41(39.8) | |  | |
| Female sex | 106(52.5) | | 62(60.2) | |  | |
| ASA classification |  | |  | | 0.763 | |
| I | 6(3.0) | | 2(1.9) | |  | |
| II | 151(74.8) | | 75(72.8) | |  | |
| III/IV | 45(22.3) | | 26(25.2) | |  | |
| Comorbidity |  |  | |  | |  |
| Hypertension | 32(15.8) | | 17(16.5) | | 0.881 | |
| Diabetes mellitus | 16(7.9) | | 15(14.6) | | 0.069 | |
| Coronary artery disease  Stroke/TIA | 3(1.5)  2(1.0) | | 2(1.9)  0(0) | | 1.000  0.551 | |
| FEV_1_/FVC, % | 104.8±7.8 | | 105.9±9.0 | | 0.319 | |
| DLCO% | 96.8±17.9 | | 96.5±18.0 | | 0.903 | |
| Chemoradiotherapy | 1(0.5) | | 0(0) | | 1.000 | |
| Tumor size, cm | 2.1±1.1 | | 2.2±1.0 | | 0.396 | |
| Clinical tumor stage |  | |  | | 0.487 | |
| T1a | 24(11.9) | | 9(8.7) | |  | |
| T1b | 99(49.0) | | 47(45.6) | |  | |
| T1c | 46(22.8) | | 28(27.2) | |  | |
| T2a | 24(11.9) | | 13(12.6) | |  | |
| T2b | 5(2.5) | | 6(5.8) | |  | |
| T3 | 3(1.5) | | 0(0) | |  | |
| T4  Advanced clinical stage (T≥2) | 1(0.5)  33(16.3) | | 0(0)  19(18.4) | | 0.643 | |

^a^ Continuous data are shown as mean ± standard deviation and categoric data as number (%). BMI: Body mass index; ASA: American Society of Anesthesiology; TIA: Transient cerebral ischemic attack; FEV_1_: Forced expiratory volume in 1 second; FVC: Forced vital capacity; DLCO: Diffusion capacity for carbon monoxide.

Supplementary table S2 Intraoperative characteristics stratified by BMI

| Variables ^a^ | 28≤BMI<30kg/m^2^  (n=202) | BMI≥30 kg/m^2^  (n=103) | *P* Value |
| --- | --- | --- | --- |
| Lymph nodes calcification | 17(8.4) | 10(9.7) | 0.707 |
| Clinical nodal involvement | 17(8.4) | 7(6.8) | 0.619 |
| Pleural adhesions | 9(4.5) | 6(5.8) | 0.601 |
| Type of resection |  |  | 0.453 |
| Segmentectomy resection | 32(15.8) | 13(12.6) |  |
| Lobectomy resection | 170(84.2) | 90(87.4) |  |
| Thoracoscopic resection |  |  | 0.067 |
| Uni-portal | 13(6.4) | 13(12.6) |  |
| Multi-portal | 189(93.6) | 90(87.4) |  |
| Approach |  |  | 1.000 |
| VATS | 196(97.0) | 100(97.1) |  |
| RATS | 6(3.0) | 3(2.9) |  |
| Anesthesia type |  |  | 0.847 |
| GA alone | 3110(83.4) | 253(83.0) |  |
| GA plus TPVB | 620(16.6) | 52(17.0) |  |
| Location of resection |  |  | 0.804 |
| Left | 75(37.1) | 31(30.1) |  |
| Left upper | 52(25.7) | 22(21.4) |  |
| Left lower | 23(11.4) | 9(8.7) |  |
| Right | 127(62.9) | 72(69.9) |  |
| Right upper | 69(34.2) | 40(38.8) |  |
| Right middle | 18(8.9) | 9(8.7) |  |
| Right lower | 40(19.8) | 23(22.3) |  |
| Ipsilateral reoperation | 1(0.5) | 0(0) | 1.000 |
| Operative time, mins | 108.9±41.7 | 107.8±36.9 | 0.816 |

^a^ Continuous data are shown as mean ± standard deviation and categoric data as number (%). BMI: Body mass index; VATS: video-assisted thoracoscopic surgery; RATS: Robotic-assisted thoracoscopic surgery; GA: General anesthesia; TPVB: Thoracic paravertebral blockade.

Supplementary **table S3 Intra- and postoperative** complications stratified by BMI

| Variables ^a^ | 28≤BMI<30kg/m^2^  (n=202) | BMI≥30 kg/m^2^  (n=103) | *P* Value |
| --- | --- | --- | --- |
| Intraoperative complications |  |  |  |
| Hypoxemia | 9(4.5) | 3(2.9) | 0.559 |
| Transfusion | 1(0.5) | 0(0) | 1.000 |
| New-onset arrhythmia | 10(5.0) | 3(2.9) | 0.554 |
| Conversion to thoracotomy | 3(1.5) | 3(2.9) | 0.668 |
| Postoperative complications |  |  |  |
| PPCs | 76(37.6) | 42(40.8) | 0.593 |
| Atelectasis | 4(2.0) | 0(0) | 0.304 |
| Pulmonary infection | 75(37.1) | 42(40.8) | 0.536 |
| Respiratory failure | 2(1.0) | 0(0) | 0.551 |
| New-onset arrhythmia | 12(5.9) | 5(4.9) | 0.696 |
| Transfusion | 2(1.0) | 0(0) | 0.551 |
| Length of hospital stay, day | 5[4-6] | 5[4-6] | 0.983 |
| 30-day readmission | 1(0.5) | 1(1.0) | 1.000 |
| Hospitalization costs, USD | 10057±2655 | 10193±2250 | 0.901 |

^a^ Continuous data are shown as mean ± standard deviation and categoric data as number (%); Length of hospital stay, values as median [interquartile range]. BMI: Body mass index; PPCs: Postoperative pulmonary complications; USD: United States dollar.
